# Novel *O*-linked methylated glycan antigens decorate secreted immunodominant glycoproteins from the intestinal nematode *Heligmosomoides polygyrus*

**DOI:** 10.1016/j.ijpara.2015.10.004

**Published:** 2016-03

**Authors:** James P. Hewitson, D. Linh Nguyen, Angela van Diepen, Cornelis H. Smit, Carolien A. Koeleman, Henry J. McSorley, Janice Murray, Rick M. Maizels, Cornelis H. Hokke

**Affiliations:** a Institute of Immunology and Infection Research, and Centre for Immunity, Infection and Evolution, School of Biological Sciences, Ashworth Laboratories, University of Edinburgh, West Mains Road, Edinburgh EH9 3JT, UK; b Department of Parasitology, Leiden University Medical Center, Albinusdreef 2, 2333 ZA Leiden, The Netherlands

**Keywords:** Nematode, *Heligmosomoides polygyrus*, Carbohydrate, Mass spectrometry, Excretory–secretory product, Antibody

## Abstract

Glycan molecules from helminth parasites have been associated with diverse biological functions ranging from interactions with neighbouring host cell populations to down-modulation of specific host immunity. Glycoproteins secreted by the intestinal nematode *Heligmosomoides polygyrus* are of particular interest as the excretory–secretory products (termed HES) of this parasite contain both heat-labile and heat-stable components with immunomodulatory effects. We used MALDI-TOF-MS and LC–MS/MS to analyse the repertoire of *N*- and *O*-linked glycans released from *Heligmosomoides polygyrus* excretory–secretory products by PNGase A and F, β-elimination and hydrazinolysis revealing a broad range of structures including novel methylhexose- and methylfucose-containing glycans. Monoclonal antibodies to two immunodominant glycans of *H. polygyrus*, previously designated Glycans A and B, were found to react by glycan array analysis to a methyl-hexose-rich fraction and to a sulphated LacDiNAc (LDN; GalNAcβ1–4GlcNAc) structure, respectively. We also analysed the glycan repertoire of a major glycoprotein in *Heligmosomoides polygyrus* excretory–secretory products, VAL-2, which contains many glycan structures present in *Heligmosomoides polygyrus* excretory–secretory products including Glycan A. However, it was found that this set of glycans is not responsible for the heat-stable immunomodulatory properties of *Heligmosomoides polygyrus* excretory–secretory products, as revealed by the inability of VAL-2 to inhibit allergic lung inflammation. Taken together, these studies reveal that *H. polygyrus* secretes a diverse range of antigenic glycoconjugates, and provides a framework to explore the biological and immunomodulatory roles they may play within the mammalian host.

## Highlights

• *Heligmosomoides polygyrus* excretory–secretory (ES) proteins carry diverse *N*- and *O*-glycans, and many are *O*-methylated.
• A methylhexose containing *O*-glycan of abundant ES glycoproteins is immunodominant.
• This dominant glycan is not the immunomodulatory heat-stable ES component.

## Graphical abstract

**Figure:**
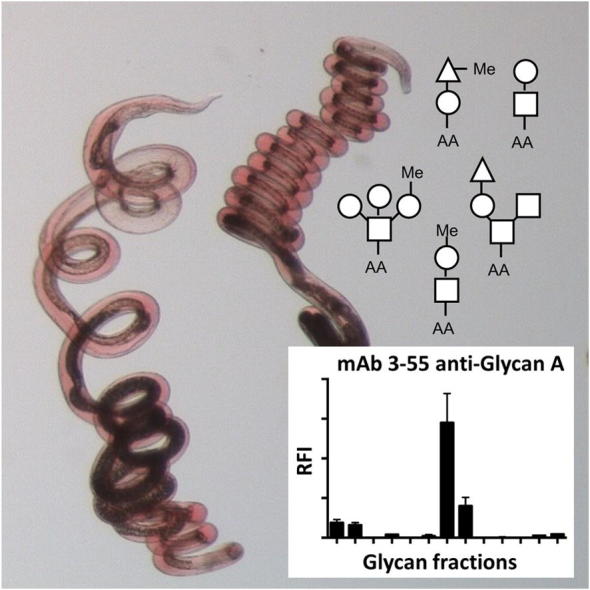

## 1 Introduction

The prominence of glycan structures in the immune recognition of parasitic helminths has been known for nearly 70 years (Campbell, 1936). Indeed, anti-carbohydrate specificities have been found to dominate the host antibody response in many different helminth infections (Omer-Ali et al., 1986, Maizels et al., 1987, Eberl et al., 2001, Kariuki et al., 2008, Hewitson et al., 2011, Paschinger et al., 2012). However, the generation of anti-glycan antibodies occurs both in susceptible hosts lacking overt anti-parasite immunity (Omer-Ali et al., 1986, Eberl et al., 2001, Kariuki et al., 2008), as well as in immunised animals resistant to infection (Vervelde et al., 2003, Kariuki et al., 2008). In some instances it is possible that glycan epitopes eliciting non-protective antibodies may even block potentially protective anti-protein responses (Dunne et al., 1987). As helminth molecules become better defined at the structural level, it is likely that the contrasting roles of specific glycans will become resolved.

Indeed, as the range and complexity of helminth-associated glycans become increasingly well-characterised, it is already clear that many specific glycans and carbohydrate motifs fulfil critical and important biological roles in the host–parasite relationship (Maizels and Hewitson, 2012, Prasanphanich et al., 2013). Most importantly, they can direct and modify the development of immunity to the benefit of the parasite (van Die and Cummings, 2010, Prasanphanich et al., 2013). This occurs through glycan binding to host pattern recognition receptors, particularly lectins such as C-type lectin receptors (CLRs) (van Die et al., 2003, van Vliet et al., 2005, Saunders et al., 2009, Meevissen et al., 2012, Klaver et al., 2013) and galectins (van den Berg et al., 2004, Breuilh et al., 2007, Burton et al., 2010), which are expressed by host innate cells such as dendritic cells (DC) and macrophages. CLR-triggered signalling pathways can both cooperate with and antagonise Toll-like receptor (TLR) signalling in helminth infection (van Liempt et al., 2007, Ritter et al., 2010, van Stijn et al., 2010a, Terrazas et al., 2013). Carbohydrate-specific interactions can further promote Th2 differentiation, as shown in the example of the schistosome ω-1 glycoprotein which enters cells through glycan binding to the mannose receptor, and subsequently subverting DC gene expression (Everts et al., 2012).

A well-studied helminth model system is that of the mouse intestinal nematode *Heligmosomoides polygyrus*, which reproduces the chronic infection pattern of both human and veterinary parasites (Reynolds et al., 2012). Infected mice mount dominant antibody responses to two distinct glycosylated antigens, termed Glycan A and Glycan B (Hewitson et al., 2011). Glycan A is an *O*-linked sugar present in the secretory products of adult parasites, termed *H. polygyrus* excretory–secretory products (HES), that are highly immunomodulatory (Grainger et al., 2010, McSorley et al., 2012, McSorley et al., 2014). Glycan A is conjugated to abundantly secreted proteins including *venom allergen/Ancylostoma* secreted protein-like (VAL)-1 and -2, which are members of a large multi-gene CAP-domain family (Pfam00188) expressed in many phyla including nematodes, cestodes and chordates (Gibbs et al., 2008, Cantacessi et al., 2009, Chalmers and Hoffmann, 2012). The Glycan A epitope is also expressed on the surface of both tissue-stage larvae and adult parasites (Hewitson et al., 2011, Hewitson et al., 2013). In contrast, Glycan B is present on a heterogeneous high molecular weight component that is highly abundant in parasite somatic tissues, as well as some glycoproteins such as those released from eggs in the intestinal lumen (Hewitson et al., 2011, Hewitson et al., 2013).

To assess the potential immunological properties of parasite glycans, both as targets of the host antibody response and as potential immunomodulators, we characterised the glycan structures within HES and investigated the structures of Glycan A and Glycan B through multiple approaches including antibody binding to glycan arrays, chemical deglycosylation and MS-based structural analysis. In addition, we analysed the glycosylation of a major glycoprotein component of HES, VAL-2 that bears Glycan A. These data reveal the range of novel structures from this helminth, including methylated fucose and hexose components that form antibody targets. Additionally, experiments with purified native VAL-2 reveal that, unlike total HES, this major glycoprotein (and by implication Glycan A) is unable to down-regulate allergic lung inflammation.

## 2 Materials and methods

### 2.1 Parasite material and antibodies

Adult HES material was collected as described elsewhere (Johnston et al., 2015). Production, purification and antigen specificity of anti-*Hp* monoclonal antibodies and generation of secondary infection immune sera were as reported previously (Hewitson et al., 2011). Native VAL-2 and VAL-3 were affinity-purified from HES using specific monoclonal antibodies (mAbs 5-S2 and 5-S1, respectively) coupled to Sepharose beads (Hewitson et al., 2011), before dialysis into PBS. ES material from adult *Nippostrongylus brasiliensis* (NES) was prepared as previously reported (Holland et al., 2000). Endotoxin levels as measured by the *Limulus* amebocyte lysate (LAL, Pierce, USA) assay were generally very low and in the range of 0.01–0.1 U/μg of protein. Silver staining was carried out as previously described (Hewitson et al., 2008).

### 2.2 ELISA

Antibody reactivity to HES and hydrogen fluoride (HF)-treated HES was performed as described previously (Hewitson et al., 2011). For competition ELISA, plate bound HES was first incubated for 30 min at 37 °C with 250 μg/ml of unlabelled mAb before addition of 10 μg/ml of biotin-labelled mAb for 2 h at 37 °C. Antibodies were biotinylated with a 20-fold molar excess of biotin using EZ-link Sulfo-NHS Biotinylation kit (Thermo Fisher Scientific, USA). Bound biotinylated antibody was detected with 1/1000 streptavidin-horseradish peroxidase (Sigma–Aldrich, USA) and developed as described by Hewitson et al. (2011).

### 2.3 Release and labelling of N-glycans from HES and VAL proteins

HES (150 μg), VAL-2 or VAL-3 (both 10 μg) were incubated with trypsin-coupled Sepharose for 16 h at 37 °C with shaking. Peptides within the supernatant were then treated with PNGase F (2 U; Roche, Germany) for 24 h at 37 °C as described previously (Borloo et al., 2013). The reaction mixture was then applied to a C_18_ reverse-phase (RP) column (150 mg; Chromabond, Macherey-Nagel, Germany), with (glycan-containing) flow-through (2 ml of 10% acetonitrile; ACN) and wash (2 ml of water) combined, partially lyophilised to remove ACN, then applied to carbon columns (150 mg; Carbograph SPE, Grace, USA). Carbon columns were washed with 6 ml of water and the PNGase F-released glycan pool eluted with 3 ml of 25% ACN and 3 ml of 25% ACN/0.05% trifluoroacetic acid (TFA), then lyophilised. Remaining peptides and glycopeptides on the C_18_ RP column were eluted with 4 ml of 30% ACN/0.1% TFA and 4 ml of 60% ACN/0.1% TFA then lyophilised. These were then resuspended in 0.1 M sodium acetate buffer pH 5 and treated with PNGase A (0.2 mU; Roche) for 24 h at 37 °C. Liberated PNGase A-sensitive glycans and (glyco)-peptides were separated as above with C_18_ RP and carbon columns. PNGase F and A-released glycan pools were resuspended in 50 μl of water, which was mixed with 50 μl of water with 2-aminobenzoic acid (anthranilic acid, AA) labelling mix (48 mg/ml 2-AA, 1 M 2-picoline-borane dissolved in 30% acetic acid/DMSO) followed by 2 h incubation at 65 °C. Labelled glycans were then cleaned up using Biogel P-10 (BioRad, The Netherlands) in 75% ACN, washed with 80% ACN, then eluted with water.

### 2.4 β-Elimination and permethylation of *O*-glycans from HES and VAL proteins

Following PNGase F and A treatment, remaining (glyco)-peptides were resuspended in 200 μl of 0.1 M NaOH/1 M NaBH_4_ and incubated for 24 h at 40 °C. Samples were neutralised on ice with 4 M acetic acid, lyophilised, then boric acid was removed by repeated evaporations (seven+) in 1% acetic acid in methanol. Released glycans were purified with C_18_ RP and carbon columns as for *N*-glycans in Section 2.3. Samples were permethylated exactly as described (Borloo et al., 2013).

### 2.5 HF treatment of HES

HES (100 μg) was dialysed into 50 mM ammonium acetate pH 7.5 and lyophilised, then twice resuspended in water and re-lyophilised, before addition of 100 μl of HF (48% v/v; Sigma) for 48 h at 4 °C as previously described (Haslam et al., 2000). Samples were then dried under nitrogen and washed twice with methanol, followed by β-elimination as described in Section 2.4.

### 2.6 Hydrazinolysis release of *O*-glycans from HES

*O*-Glycans were released from lyophilised HES (500 μg) using the Ludger Liberate Hydrazinolysis Glycan Release Kit (Ludger, United Kingdom) according to the manufacturer’s instructions (6 h, 60 °C). Sample acidification (0.1% TFA) during the release procedure prevents glycan peeling/degradation (Kozak et al., 2012). *O*-Glycans were then labelled with AA as described for *N*-glycans in Section 2.3.

### 2.7 Glycan arrays

Hydrazinolysis-released *O*-glycans were separated by RP-HPLC as described previously (van Diepen et al., 2012a) with flow rate 500 μl/min and initial conditions: buffer A 0.1% formic acid/water (10 min) followed by 0–100% gradient buffer B (0.1% formic acid/95% ACN; 30 min) and 10 min isocratic elution. Fractions (F) were collected manually based on AA fluorescent signal (F1–44). AA-labelled glycans could only be detected in F14–26 by LC–MS/MS (data not shown). Glycan concentrations within each fraction were estimated by comparison with a known AA-labelled standard glycan (H_5_N_2_-AA, data not shown). Fractions were dissolved in 20 μl of 1× spotting buffer (Nexterion Spot; Schott Nexterion, Germany) with 10% DMSO in 384-well V-bottom plates (Genetix, USA) at half log dilutions 0.03–10 μM. Samples (five of each) were printed on epoxysilane-coated glass slides (Slide E, Nexterion) and probed with mAb (10 μg/ml) or polyclonal mouse serum (1/100), and analysed as before (van Diepen et al., 2012a), with secondary antibodies Alexa-Fluor 555-labelled goat anti-mouse IgM and Alexa-Fluor 647-labelled goat anti-mouse IgG (both 1/1000; Life Technologies, USA). Anti-HES mAbs were also tested against a mammalian glycan array (v5.1) by Core H of the Consortium for Functional Glycomics (details available at http://www.functionalglycomics.org/static/consortium/resources/resourcecoreh17.shtml). Arrays were probed as described elsewhere (van Stijn et al., 2010b).

### 2.8 MALDI-TOF(/TOF) MS and LC–MS analysis

Glycans were analysed as described (Smit et al., 2015) with an Ultraflex II MALDI-TOF mass spectrometer (Bruker Daltonics, Germany) in negative ion (AA labelled PNGase F and A-released *N*-glycans) or positive ion (permethylated β-elimination-released *O*-glycans) reflectron mode with 2,5-dihydroxy benzoic acid (DHB) (20 mg/ml in 20% ACN) as matrix. *N*-glycans were eluted directly onto target plates with matrix following Zip-Tip C_18_. MS spectra were annotated using Glyco-Peakfinder (http://www.glyco-peakfinder.org/). For LC–MS analysis, glycans were applied to a RP column (PepMap, 3 μm × 75 μm × 100 mm) using an Ultimate 3000 nano-LC system (both Dionex/LC Packings) at room temperature. The column was equilibrated with eluent A (0.1% formic acid/water) at flow rate 200 nl/min. Following sample injection, conditions were changed to 10% solvent B (0.1% formic acid/95% ACN) with a gradient to 60% B over 45 min, then isocratic elution for 10 min. The LC column was coupled to an Esquire HCT Ultra ESI-IT-MS (Bruker Daltonics) equipped with an online nanospray source in positive-ion mode. Conditions were as in Borloo et al. (2013). Ions from *m*/*z* 300–1800 and 140–2200 were registered in MS and MS/MS mode, respectively. MS/MS spectra were manually interpreted using Bruker Daltonics DataAnalysis software (Bruker Daltonics).

### 2.9 OVA airway allergy model and mice

Induction of airway inflammation was carried out in BALB/c female mice as previously described (McSorley et al., 2012, McSorley et al., 2015) with HES (5 μg) or VAL-2 (2 μg) added as soluble proteins to a suspension of alum-precipitated ovalbumin (OVA), and injected i.p. in PBS on days 0 and 14. Heat inactivation of proteins was carried out at 100 °C for 20 min. At 28, 29 and 30 days, mice were challenged with ovalbumin in the airways and at day 31 lung tissue and bronchioalveolar lavage (BAL) cells were analysed by flow cytometry for CD45.2^+^CD11b^+^SiglecF^+^SS^hi^ eosinophil infiltration as a marker of allergic inflammation (McSorley et al., 2012). All animal protocols adhered to the guidelines of the UK Home Office, complied with the Animals (Scientific Procedures) Act 1986, were approved by the University of Edinburgh Ethical Review Committee, and were performed under the authority of the UK Home Office Project Licence number 60/410.

## 3 Results

### 3.1 Glycan array analysis indicates that Glycan A is a novel structure, whilst Glycan B is related to sulphated glycosaminoglycan molecules

Infection with *H. polygyrus* elicits an immunodominant antibody response against two glycan targets defined as Glycans A and B (Hewitson et al., 2011). Glycan A is an *O*-linked sugar coupled to multiple carrier proteins, most prominently members of the CAP superfamily of glycoproteins (Pfam00188) which includes several VAL antigens. Glycan B is present on high molecular weight species which migrate diffusely on SDS–PAGE as well as a ∼65-kDa molecule distinct from the VAL proteins (Hewitson et al., 2011).

Panels of mAbs directed against Glycans A and B were used to screen an extensive glycan array (Consortium for Functional Glycomics (CFG), v5.1) containing 610 native and synthetic sugars, predominantly related to mammalian glycosylation. Similar screening has previously been performed with polyclonal antisera against the related parasites *Haemonchus contortus* (van Stijn et al., 2010b) and *Trichinella spiralis* (Aranzamendi et al., 2011), as well as mAbs to tumour-associated epitopes (Noble et al., 2013, Chua et al., 2015). First, three IgM anti-Glycan A mAbs (mAbs 13.1, 3–42 and 3–55) were selected as showing the highest binding affinity to HES by ELISA from amongst 12 available antibodies (Supplementary Fig. S1). These mAbs, however, did not bind to any of the glycans on the array (data not shown). Anti-Glycan A mAbs also failed to react with ES material obtained from the closely-related rat nematode, *N. brasiliensis* (data not shown), suggesting that Glycan A is a novel structure specific to *H. polygyrus*.

**Fig. 1:**
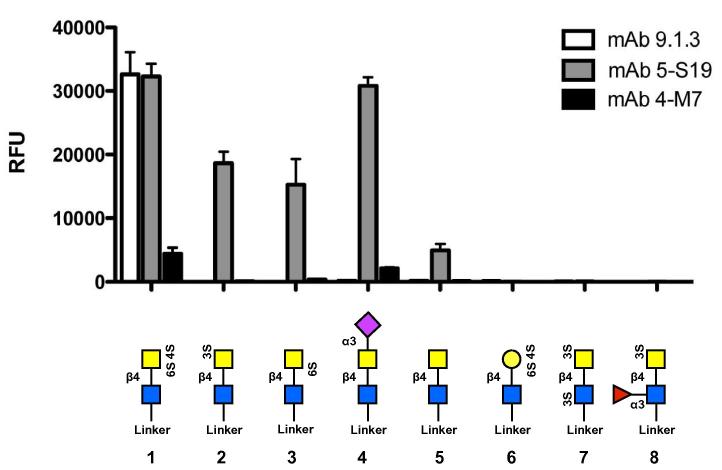
Characterisation of anti-Glycan B monoclonal antibody specificity. Binding of three distinct anti-Glycan B monoclonal antibodies (clones 9.1.3, 5-S19 and 4-M7) to the Consortium of Functional Glycomics mammalian glycan array v5.1 (details available at http://www.functionalglycomics.org/static/consortium/resources/resourcecoreh17.shtml). RFU, relative fluorescent units. Error bars indicate the standard deviation of the mean of six replicates. Monosaccharide composition and linkages as indicated. Dark filled square, GlcNAc; light filled square, GalNAc; filled circle, galactose; filled triangle, fucose; filled diamond, sialic acid (NeuAc).

In contrast to anti-Glycan A mAbs, all three anti-Glycan B mAbs tested (mAbs 9.1.3, 5-S19 and 4-M7 (Hewitson et al., 2011)) showed significant binding to several related structures (Fig. 1). Maximal binding of each mAb was seen to the disulphated LDN structure (6S)(4S)GalNAc(β1–4)GlcNAc. Whilst mAb 9.1.3 showed a strong preference for this glycan, 5-S19 also bound the related structures Neu5Ac(α2–3)GalNAc(β1–4)GlcNAc (as did 4-M7) and monosulphated (3S)- and (6S)-GalNAc(β1–4)GlcNAc, indicating they target GalNAc residues with negatively charged substitutions at positions 3, 4 and 6. mAb 5-S19, but neither of the other antibodies, has a weak affinity for unsubstituted LDN. It is also important that the terminal residue is GalNAc, since no binding was detected to (6S)(4S)Gal(β1–4)GlcNAc. Finally, substitution of the core GlcNAc residue in (3S)GalNAc(β1–4)GlcNAc at position 3, with either (3S) or fucose, prevented binding. Together, this suggests that anti-Glycan B mAbs bind to negatively-charged LDN motifs, with one potential target being sulphated glycosaminoglycans (GAG).

### 3.2 Mass spectrometric characterisation of ES glycans

**Fig. 2:**
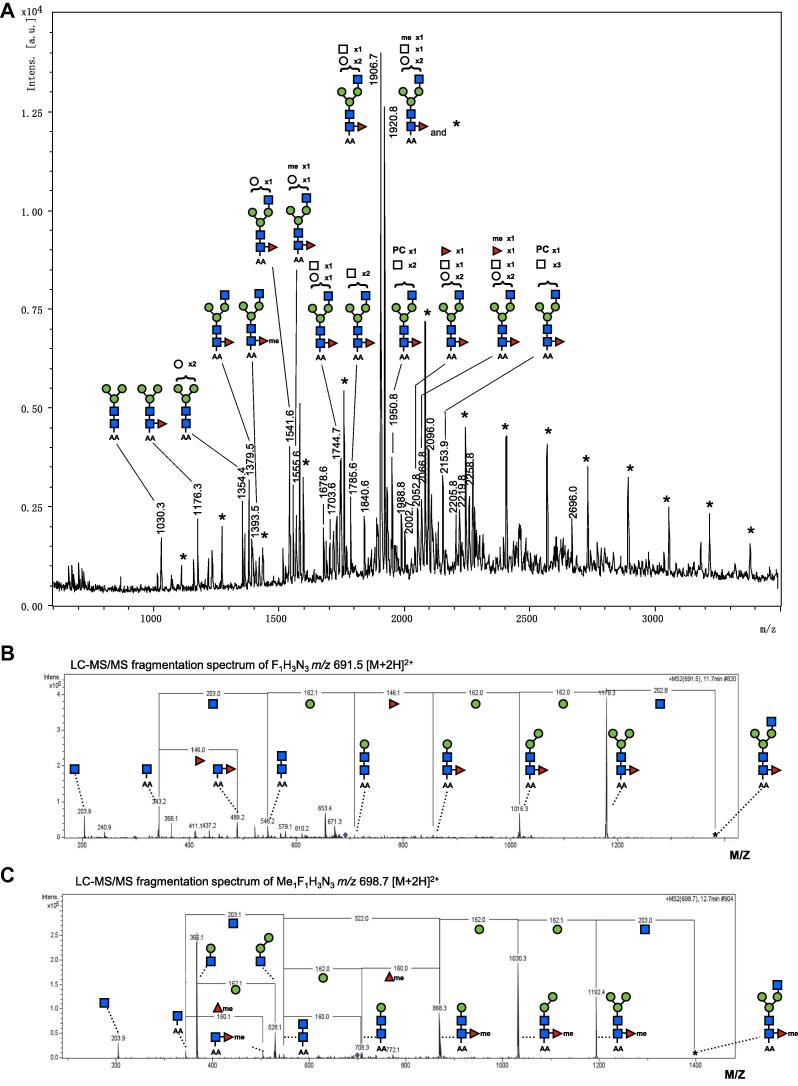
Profiles of *N*-linked glycans released by PNGase-F treatment from *Heligmosomoides polygyrus* excretory–secretory products (HES). (A) MALDI-TOF-MS of *N*-linked glycans released from *Heligmosomoides polygyrus* excretory–secretory products by treatment with PNGase F. Glycans were labelled with anthranilic acid and detected in the negative ion reflectron mode as [M−H]^−^. Structural assignments on the basis of monosaccharide composition and *N*-glycan biosynthetic rules were supported by LC–MS/MS fragmentation data. The signals marked with ^∗^ indicate a contaminating hexose ladder often seen in glycan preparations from diverse sources (Aoki et al., 2008, Talabnin et al., 2013). Filled square, GlcNAc; filled circle, mannose; filled triangle, fucose; open square, HexNAc, open circle, hexose; me, methylated monosaccharide; PC, phosphorylcholine. (B–C) LC–MS/MS fragmentation spectra of the glycans detected at *m*/*z* 1379.5 and 1393.5 [M−H]^−^ ion species in (A). Fragmentation of *m*/*z* 691.5 (B) and *m*/*z* 698.7 (C) [M+2H]^2+^ double charged ion-species is indicative of the presence of a methylated core-linked fucose in (C). ^∗^, parent ion.

Previously, we showed that both Glycans A and B are predominantly conjugated through *O*- rather than *N*-linked glycosylation, and that antibodies to these glycans dominate the humoral response of infected mice (Hewitson et al., 2011). In preparing HES glycoproteins for MS analysis, therefore, we first liberated all *N*-linked sugars through sequential treatment with PNGase F and PNGase A, prior to reductive β-elimination to release *O*-linked structures*.* This procedure also offered the opportunity to characterise *N*-linked structures through MALDI-TOF-MS. We thereby identified typical *N*-glycan structures released both by PNGase F (Fig. 2A) (often with core α1,6-linked fucose) and, to a very limited extent, by PNGase A (with additional core α1,3-linked fucose (not shown)). *N*-Glycan structures extended with multiple hexose and *N*-acetylhexosamine residues were detected, some of which appear to contain a phosphorylcholine (PC) substitution as previously suggested to be present in HES (Hewitson et al., 2011). Closer inspection of the MALDI-TOF-MS revealed several sets of peaks with mass differences of 14 Da, indicating the possible occurrence of methylated glycans. These sets of glycan-ions with a mass difference of 14 Da were confirmed by LC–MS analysis and fragmentation of selected parent ions (e.g. *m*/*z* 691.5 [M+2H]^2+^ (F_1_H_3_N_3_) and *m*/*z* 698.7 [M+2H]^2+^ (meF_1_H_3_N_3_); F, deoxyhexose/fucose; H, hexose; *N*,*N*-acetylhexosamine; meF, methylated fucose; Fig. 2B, C) reveals that this mass difference is due to the presence of a 160 Da monosaccharide in place of the core fucose (146 Da), consistent with an *O*-methylated fucose residue, as previously reported in some other nematode glycans (Khoo et al., 1991, Wohlschlager et al., 2014).

### 3.3 Mass spectrometric characterisation of *O*-glycans present in HES

**Fig. 3:**
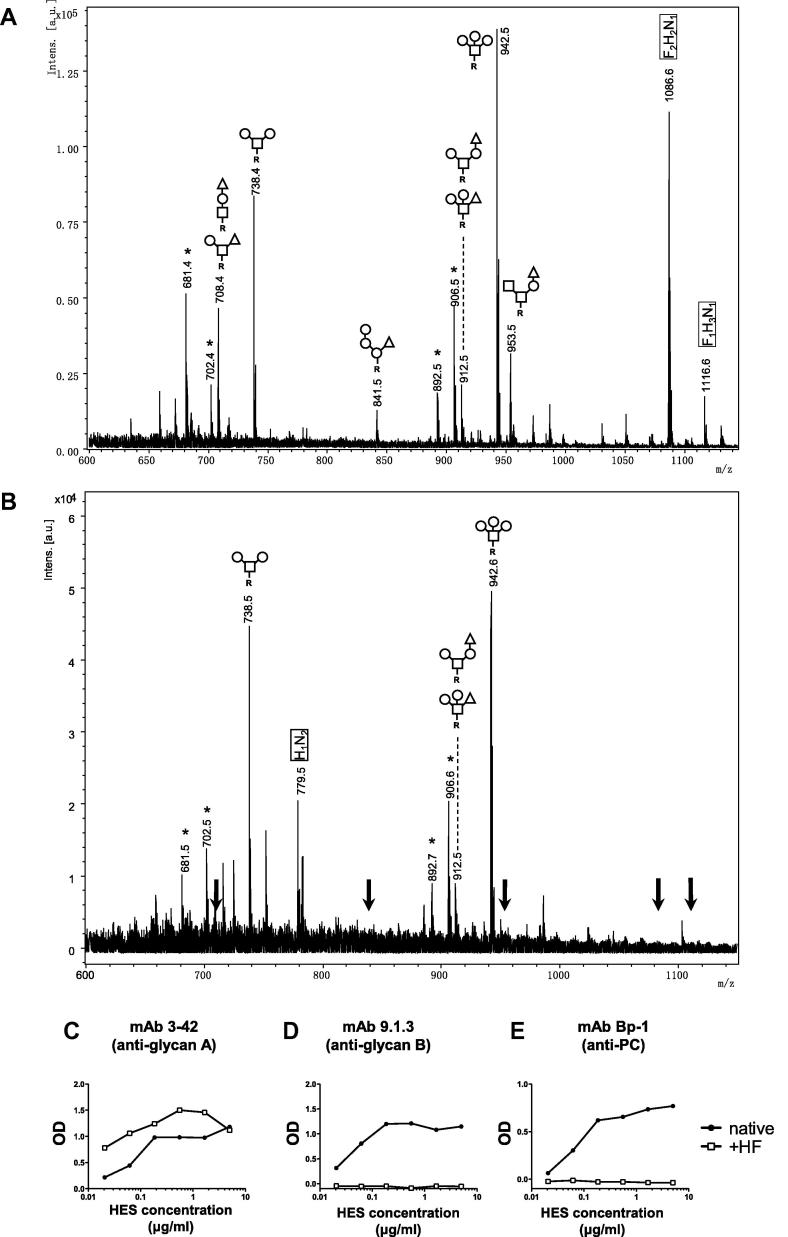
Profiles of *O*-glycans released by reductive β-elimination from *Heligmosomoides polygyrus* excretory–secretory products (HES). MALDI-TOF-MS of permethylated (A) *Heligmosomoides polygyrus* excretory–secretory products and (B) hydrogen fluoride-treated *Heligmosomoides polygyrus* excretory–secretory products *O*-glycans released by β-elimination and detected in positive ion reflectron mode as [M+Na]^+^. Proposed compositions (HexNAc, open square; hexose, open circle; deoxyhexose, open triangle; R, reduced end) and arrangements are based on LC–MS/MS fragmentation spectra (e.g. Supplementary Fig. S2). Unidentified peaks not matching with glycan compositions are marked with ^∗^. Compositions in boxes without proposed structure produced ambiguous LC–MS/MS fragmentation patterns (F, deoxyhexose/fucose; H, hexose; *N*,*N*-acetylhexosamine). Arrows in (B) represent deoxyhexose (fucose)-containing glycans missing in hydrogen fluoride-treated *Heligmosomoides polygyrus* excretory–secretory products. (C–E) ELISA reactivity of (C) anti-Glycan A monoclonal antibody clone 3–42 (similar results with monoclonal antibody 13.1 and 2–13, data not shown), (D) anti-Glycan B monoclonal antibody clone 9.1.3, and (E) anti-phosphorylcholine monoclonal antibody clone Bp-1 to native or hydrogen fluoride-treated *Heligmosomoides polygyrus* excretory–secretory products.

We then analysed total HES *O*-glycans released by reductive β-elimination. Released *O*-glycans were permethylated followed by purification using liquid–liquid extraction to permit analysis by MALDI-TOF-MS and LC–MS/MS, which indicated that HES contains a relatively complex pattern of at least nine small *O*-linked glycans with two to five monosaccharide units (Fig. 3A). Predominant were an unusual tetrasaccharide consisting of a reduced HexNAc residue tri-substituted with terminal hexoses at *m*/*z* 942.5 [M+Na]^+^ (supporting LC–MS/MS fragmentation spectrum is shown in Supplementary Fig. S2), and the di-substituted variant at *m*/*z* 738.4 [M+Na]^+^. A similarly abundant peak was observed at *m*/*z* 1086.6 [M+Na]^+^, which equates to a composition of F_2_H_2_N_1_, likely representing the di-fucosylated variant(s) of the glycan species at *m*/*z* 738.4 and 912.5 [M+Na]^+^.

Next, we analysed by MS the permethylated *O*-glycans liberated from HF-treated HES. HF cleaves off α1,3-linked fucose, as well as other relatively labile moieties such as PC (Haslam et al., 2000). MALDI-TOF-MS ascertained that HF treatment caused the complete loss of the dominant deoxyhexose-containing peak at *m*/*z* 1086.6 (F_2_H_2_N_1_), as well as the minor deoxyhexose-containing glycan species observed at *m*/*z* 953.5 (F_1_H_1_N_2_), 841.5 (F_1_H_3_), and 708.4 (F_1_H_1_N_1_), with the emergence of *m*/*z* 779.5 (H_1_N_2_), possibly derived from *m*/*z* 953.5 (F_1_H_1_N_2_) (Fig. 3B) (all ions observed as [M+Na]^+^). In contrast, HF treatment left the dominant peaks of *m*/*z* 942.5 (H_3_N_1_) and 738.4 (H_2_N_1_), the latter with increased relative intensity due to defucosylation of F_1-2_H_2_N_1._ Only a single, minor signal was left of a glycan species with a deoxyhexose residue (*m*/*z* 912.5 (F_1_H_2_N_1_) after HF treatment. Possibly, this signal remains due to incomplete removal of both fucoses from the major F_2_H_2_N_1_ glycan. These observations suggest that most deoxyhexoses in HES *O*-glycans are α1,3-linked fucose and not α1,4/6-linked fucose (Haslam et al., 2000), although it is not clear how sensitive α1,2-linked fucose, if present, would be to HF.

To investigate whether HF sensitive fucose residues form part of antigenic motifs in HES we also tested the immunological reactivity of HES following HF treatment. Binding of anti-Glycan A mAbs is actually enhanced following HF treatment (Fig. 3C), suggesting that the target epitope may be one of the HF-resistant structures described. In contrast, binding of anti-Glycan B mAb 9.1.3 which targets sulphated LDN (Fig. 1) was completely ablated (Fig. 3D), consistent with the reported effects of HF to remove side chains from GAGs such as chondroitin sulphate (Olson et al., 1985). In addition, we tested binding of an antibody to PC, a common modification of nematode sugars (Houston and Harnett, 2004), using the mAb Bp-1 (Sutanto et al., 1985), and confirmed that whilst this epitope is present in HES, it is also completely removed by HF (Fig. 3E). The PC-containing target of mAb Bp-1 and the sulphate-substituted LDN Glycan B motif are apparently not present or present in only undetectable relative amounts amongst the HES *O*-glycans detected in our MS analyses.

### 3.4 Structure and antigenicity of hydrazinolysis-released, non-permethylated, HES *O*-glycans

To be able to further characterise the *O*-glycan target of anti-Glycan A mAb we next employed a recently described approach to determining antigenic glycans, in which they are isolated, labelled, fractionated and printed onto solid-phase arrays for probing with antibody reagents (van Diepen et al., 2012b, van Diepen et al., 2015). *O*-glycans liberated by β-elimination are unsuitable for this purpose as their reducing end is converted to a non-reactive alditol, and permethylation modifies the native structure to the extent it would likely prevent antibody binding. In addition, in view of the indications that methylated glycans are present amongst the HES *N*-glycans (Fig. 2A–D), structural investigation of intact unmodified *O*-glycans allows the identification of methyl-substitutions that would be obscured by permethylation. *O*-glycans were therefore released from HES through hydrazinolysis, using conditions that favour *O*-glycan release, followed by *N*-acetylation, resulting in free glycans with an intact reducing end sugar. These were then labelled at the reducing sugar with AA, fractionated, and analysed by LC–MS/MS prior to printing on a solid support (van Diepen et al., 2015).

**Fig. 4:**
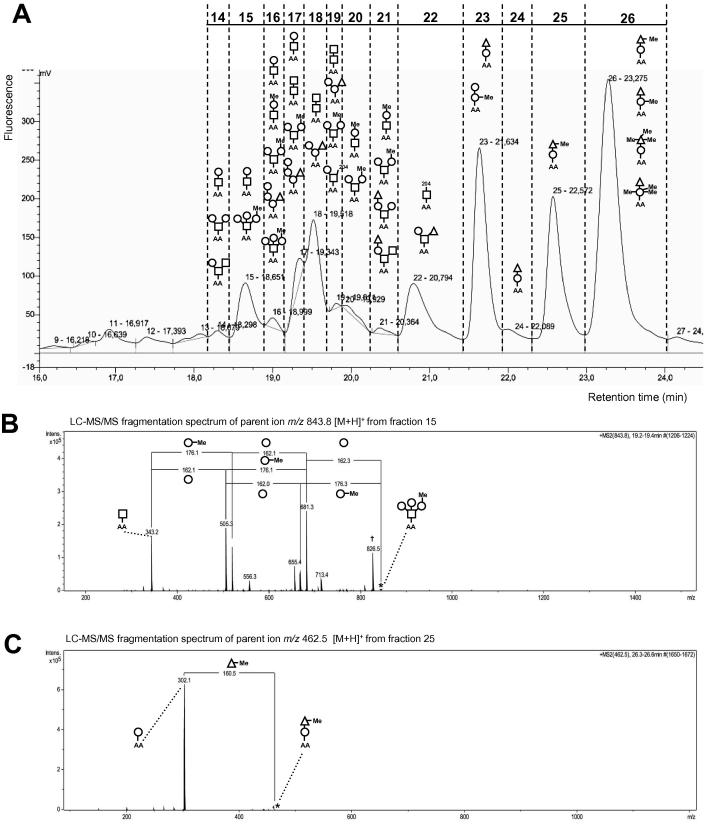

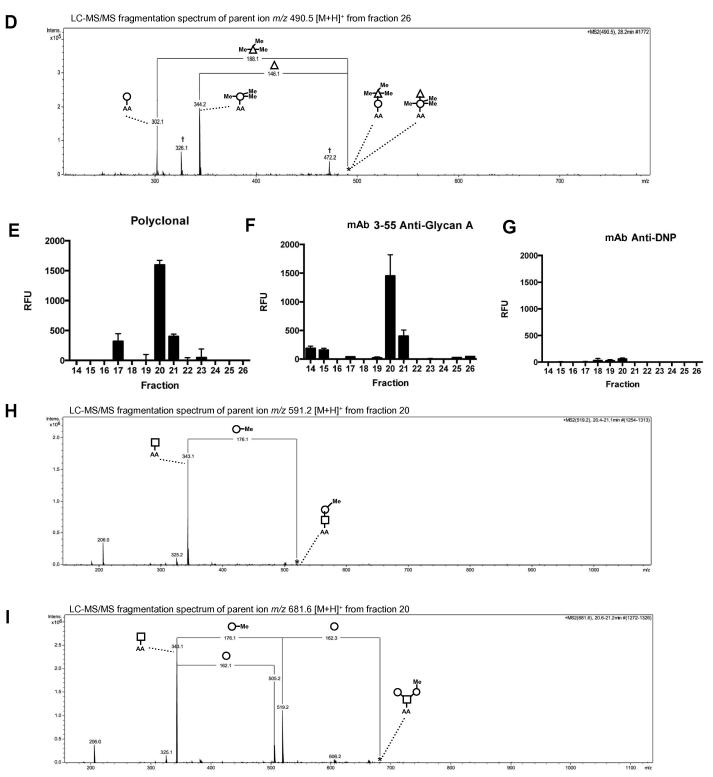
Antibody reactivity to reverse phase LC-fractions of hydrazinolysis-released *O*-glycans of *Heligmosomoides polygyrus* excretory–secretory products (HES). (A) Reverse phase-chromatogram of anthranilic acid labelled *O*-glycans released from *Heligmosomoides polygyrus* excretory–secretory products by hydrazinolysis. Fractions of anthranilic acid labelled glycans were manually collected following reverse phase-LC separation and individual fractions analysed by LC–MS/MS (B–D, H–I) and MALDI-TOF-MS/MS (data not shown) for glycan fragmentation signatures (anthranilic acid-labelled HexNAc, hexose or deoxyhexose, or methylated variants thereof). Proposed compositions are shown (HexNAc, open square; hexose, open circle; deoxyhexose, open triangle; Me, methylated monosaccharide; AA, anthranilic acid. (B) Fragmentation of glycan *m*/*z* 843.8 [M+H]^+^ found in LC-fractions 15 and 16. Loss of mass 176 (hexose 162 + 14 Da) is suggestive of single methylation of hexose in meH_1_H_2_N_1_-anthranilic acid, with core HexNAc (203 + 140 anthranilic acid label = 343). † Represents loss of water (18 Da). (C) Fragmentation of glycan *m*/*z* 462.5 [M+H]^+^ found in LC-fractions 25 and 26. Loss of mass 160 (fucose 146 + 14 Da) suggests fucose methylation in meF_1_H_1_-anthranilic acid with core hexose (162 + 140 anthranilic acid label = 302). (D) Fragmentation of glycan *m*/*z* 490.5 [M+H]^+^ found in LC-fraction 26 is consistent with two distinct glycan structures. Core hexose (162 + 140 anthranilic acid label = 302) bound to potential trimethylated fucose (146 + 14 + 14 + 14 = 188) and core trimethylated hexose (162 + 140 anthranilic acid label + 14 + 14 + 14) bound to fucose (146). †, loss of water (18 Da). (E–G) Antibody reactivity to glycan-containing LC-fractions 14–26. (E) Secondary infection IgG, (F) anti-glycan A IgM monoclonal antibody 3–55, (G) anti-DNP IgM isotype control monoclonal antibody. (H–I) Fragmentation spectra of glycans (H) *m*/*z* 591.2 [M+H]^+^ and (I) *m*/*z* 681.6 [M+H]^+^ found in LC-fraction 20, which reacts with anti-Glycan A monoclonal antibody.

RP fractionation of the labelled hydrazinolysis-released *O*-glycan repertoire, followed by LC–MS/MS analysis of each fraction revealed a complex peak pattern with multiple glycan structures (Fig. 4A, Table 1). Together, this analysis of hydrazinolysis-released glycans revealed several key new insights. First, stoichiometric labelling at the reducing end combined with LC and fluorescence detection provides a more accurate quantification of different glycans than can be obtained from MALDI-TOF-MS, where ion suppression prevents reliable observation of the small di- and trisaccharide glycans. LC (Fig. 4A) and LC–MS/MS (Fig. 4B–D) reveal that the most abundant glycan structures released by hydrazinolysis are disaccharides, including H_1_N_1_-AA (fractions 14–17), N_2_-AA (fractions 17–19) and F_1_H_1_-AA (fractions 23–24).

Secondly, whilst there was good concordance between most abundant glycan species identified following β-elimination and hydrazinolysis (i.e. F_1_H_1_N_1_-AA, H_2_N_1_-AA, F_1_H_3_-AA, F_1_H_2_N_1_-AA, F_1_H_1_N_2_-AA; Table 1), the latter did not yield the H_3_N_1_-AA species (equivalent to β -elimination *m*/*z* 942.5 [M+Na]^+^). Instead, fractions 15–16 contained a glycan with the putative structure of meH_1_H_2_N_1_-AA, where meH represents a methylated hexose residue, based on LC–MS/MS fragment differences of 176 Da (hexose + 14) (Fig. 4B), confirming that similar to HES, *N*-glycans the *O*-glycans contain methylated species. We discount the possibility of this mass representing a hexuronic acid residue (U) as earlier analysis of permethylated β-elimination material detected H_3_N_1_-AA but no U_1_H_2_N_1_-AA structures (Fig. 3). Interestingly, several *O*-glycan species contain a hexose residue at the reducing end (Table 1). This is uncommon as *O*-glycans are normally based on HexNAc. Specific degradation during hydrazinolysis of Galβ1–3GalNAc core types has been observed, leading to the formation of a reducing hexose residue in schistosome *O*-glycan samples (Van Diepen et al., 2015). However, hexose-based glycans were observed also in the β-elimination-released *O*-glycan pool suggesting that degradation during hydrazinolysis is not their primary source.

Further inspection revealed a number of other methyl-hexose containing glycan structures, including meH_1_N_1_-AA (fractions 20–21), meH_1_H_1_N_1_-AA (fractions 16–17, 19–21), meH_1_H_1_-AA (fraction 23) and F_1_meH_1_-AA (fraction 26). Moreover, a methyl-fucosylated species meF_1_H_1_-AA (fractions 25–26) was deduced from the LC–MS/MS spectrum of *m*/*z* 462.5 [M+H]^+^, indicating the presence of the 160-Da methylated fucose monosaccharide (Fig. 4C). Methylated sugars behaved as expected during RP fractionation, eluting at higher concentrations of organic solvent than their non-methylated equivalent, reflecting their increased hydrophobicity (Fig. 4A). Finally, the loss-of-mass observations of 204 Da (162+14+14+14) and of 188 Da (146+14+14+14), and the presence of the *m*/*z* 344 fragment ion (trimethyl hexose + AA label) in LC–MS/MS spectra of fractions 19, 22 and 26 are suggestive of the presence of trimethylated hexose and fucose residues, respectively (Fig. 4D).

We then probed the printed RP-LC fractions with both polyclonal and mAb reagents. Polyclonal antibodies were collected from mice rendered immune by sequential *H. polygyrus* infection, drug-induced clearance, and secondary challenge infection (McCoy et al., 2008, Hewitson et al., 2011), and were highly reactive to HES but not to the complex glycosylated egg antigens (SEA) from *Schistosoma mansoni* (data not shown). Both IgG (Fig. 4E) and IgM (data not shown) antibodies showed preferential reactivity with fraction 20 comprising putative methylhexose-containing di- and trisaccharides; no signal was seen with naïve mouse sera (data not shown). Furthermore, we found some anti-Glycan A IgM antibodies (mAb 2–13, 2–62, 3–29 and 3–55) bound to fraction 20 (Fig. 4F and data not shown), whilst a control IgM mAb did not (anti-DNP; Fig. 4G), indicating that methylated hexose is the target of anti-Glycan A mAb 2–13, 2–62, 3–29 and 3–55. A mAb directed against Glycan B (mAb 9.1.3) was unreactive to the array (data not shown).

Fragmentation analysis of the antibody-reactive fraction 20 by MS/MS revealed two principal ion species, *m*/*z* 591.2 (Fig. 4H) and *m*/*z* 681.6 (Fig. 4I) [M+H]^+^. Both contain methylated hexoses and the latter corresponds to the *m*/*z* 738.6 [M+Na]^+^ ion of the H_2_N_1_ species observed in permethylated HF-treated HES which retained antibody binding (Fig. 3B). The meH_1_H_1_N_1_ *O*-glycan consequently represents a strong candidate for the identity of Glycan A.

### 3.5 Marked heterogeneity in *O*-glycans on the VAL-2 antigen

**Fig. 5:**
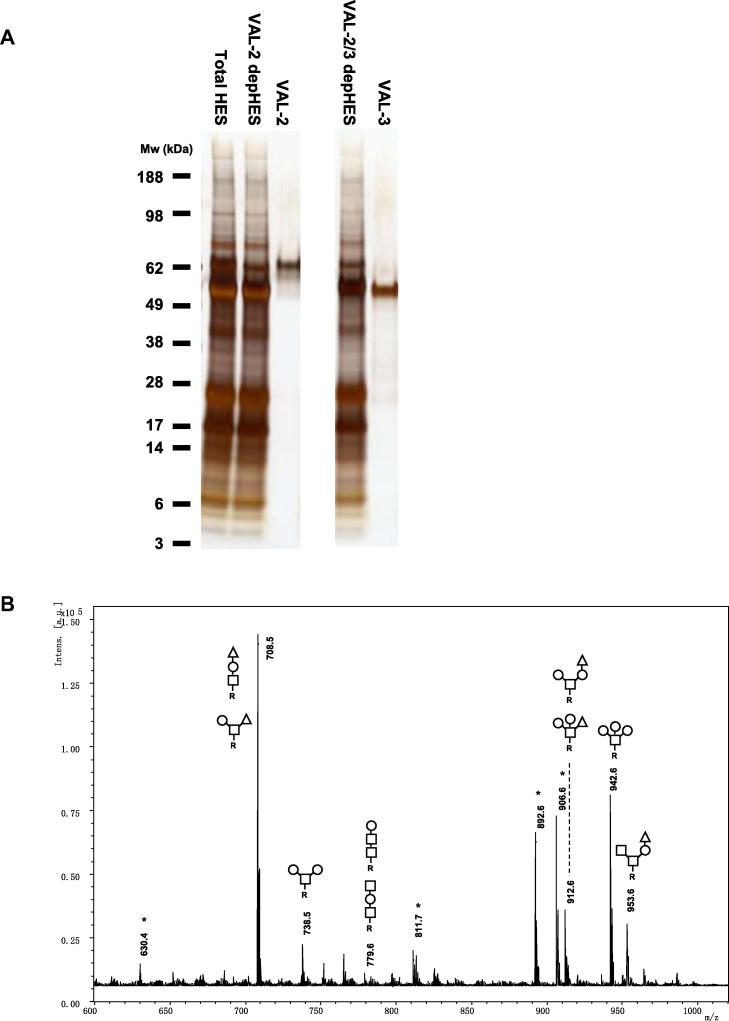
Profiles of venom allergen/*Ancylostoma* secreted protein-like (VAL-2) *O*-glycans released by reductive β-elimination. (A) One-dimensional silver stained SDS–PAGE of *Heligmosomoides polygyrus* excretory–secretory products (HES), VAL-2-depleted *Heligmosomoides polygyrus* excretory–secretory products, native VAL-2, VAL-2 and 3-depleted *Heligmosomoides polygyrus* excretory–secretory products and native VAL-3. Mw markers (kDa) are indicated. (B) MALDI-TOF-MS of permethylated VAL-2 glycans released by β-elimination and measured as in Fig. 3. HexNAc, open square; hexose, open circle; deoxyhexose, open triangle; R, reduced end.

To complement our analysis of total HES *O*-glycans, we also analysed glycosylation of a single protein component of HES, the Glycan A-bearing glycoprotein VAL-2, a member of the CAP domain protein family (Pfam00188). This was affinity purified from HES using a specific mAb, alongside the related VAL-3 protein that lacks Glycan A (Fig. 5A; (Hewitson et al., 2011)). PNGase F- and PNGase A-treated VAL-2 glycopeptides from each protein were subjected to β-elimination and MALDI-TOF-MS. Despite VAL-2 and VAL-3 each containing a single predicted *N*-glycosylation site (N340GS and N143LS, respectively), in neither case did we detect PNGase F- or A-released *N*-glycans by MALDI-TOF-MS (data not shown). Permethylated *O*-glycans liberated from VAL-2 by β -elimination were readily detected (Fig. 5B), revealing similarly heterogeneous *O*-glycan modifications to those observed in total HES (Fig. 3A), with *m*/*z* 708.5 (F_1_H_1_N_1_) particularly prominent and *m*/*z* 942.6 (H_3_N_1_) also strongly represented. In all, 10 distinct structures were determined by LC–MS/MS, including one with *m*/*z* 779.6 (H_1_N_2_) that was not observed in total HES. Other differences to total HES *O*-glycans included the complete absence from VAL-2 of the *m*/*z* 1086.6 (F_2_H_2_N_1_) species, confirming this structure is not Glycan A, a conclusion already supported by the HF-sensitivity of this glycan (Fig. 3B). In contrast to VAL-2, except for signals with a very low signal-to-noise ratio representing H_2_N_1_ (*m*/*z* 738.6, [M+Na]^+^) and H_1_N_1_ (*m*/*z* 512.3 [M+H]^+^), no *O*-linked glycans were detected in VAL-3 (data not shown), which is consistent with VAL-3 having very few serine or threonine residues predicted to be *O*-glycosylated (Hewitson et al., 2011).

### 3.6 Glycan A is not responsible for the immunomodulatory effects of HES

HES is able to both inhibit in vitro DC activation in response to inflammatory stimuli (Segura et al., 2007, Massacand et al., 2009) and in vivo allergic lung inflammation following allergen sensitisation (McSorley et al., 2012). In both instances, these effects have been reported to be resistant to heat denaturation, suggesting a role for parasite glycans. To investigate whether (heat-stable) Glycan A was responsible for these effects, we initially determined whether native or heat-denatured Glycan A-bearing VAL-2 was able to inhibit LPS-dependent production of IL-12p70 by bone-marrow derived DCs (BMDCs). However, in contrast to an earlier report (Massacand et al., 2009) we found that the inhibitory effect of HES was completely ablated when HES was heat-treated, and that native Glycan A-bearing VAL-2 was similarly unable to modulate DC activation (data not shown).

**Fig. 6:**
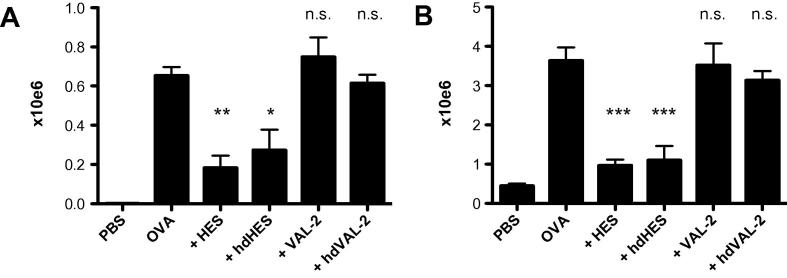
Venom allergen/*Ancylostoma* secreted protein-like antigen (VAL-2) does not inhibit allergic lung inflammation. Ovalbumin in alum adjuvant (OVA) airway inflammation was induced as detailed in Section 2.9 with *Heligmosomoides polygyrus* excretory–secretory products (HES), heat-denatured (hd) *Heligmosomoides polygyrus* excretory–secretory products, native VAL-2 or hdVAL-2 delivered during sensitisation. On day 31 after first sensitisation, bronchioalveolar lavage (A) and lung tissue (B) eosinophil numbers were determined by fluorescence-activated cell sorting. Values represent the mean of four mice per group ± S.E.M. Significance was determined by ANOVA compared with the ovalbumin in alum adjuvant group, or as indicated. ^*^P < 0.05, ^**^P < 0.01, ^***^P < 0.001, not significant (n.s.; *P* > 0.05).

We did, however, find that in accordance with our previous publication (McSorley et al., 2012), heat-treated HES retained immunosuppressive activity in a murine allergy model. Thus, treatment of mice with HES during immunization with ovalbumin in alum adjuvant significantly inhibited eosinophilia in both BAL (Fig. 6A) and lung tissue (Fig. 6B), and this effect was replicated with heat-denatured HES. However, an equivalent treatment regime with purified VAL-2 (native or heat-denatured) failed to reduce eosinophil numbers. Together, this suggests that whilst parasite glycans may be able to inhibit alum-dependent sensitisation to allergic stimuli, this is not due to either Glycan A or indeed any of the other *O*-glycans linked to VAL-2.

## 4 Discussion

Glycans play a critically important part in the ability of parasites to interact with their chosen host, and in the manner in which the host immune response to infection unfolds (Nyame et al., 2004, Hokke et al., 2007, Harn et al., 2009, van Die and Cummings, 2010, Prasanphanich et al., 2013). The murine intestinal nematode *H. polygyrus* is a widely studied model of chronic helminth infection in which host immunity is profoundly down-modulated by a range of parasite secreted products, termed HES (Hewitson et al., 2009, Reynolds et al., 2012). We now show that the HES molecules include a wide range of glycan formations including several methylhexose and methylfucose conjugates that represent novel structures, and two glycosylated targets recognised by mAbs generated in infected mice.

The host immune response to *H. polygyrus* is dominated by antibodies to glycan specificities in the secreted products, HES (Hewitson et al., 2011). Whilst class-switched IgG1 antibodies are essential mediators of immunity to gut nematodes (McCoy et al., 2008, Liu et al., 2010, Hewitson et al., 2015), the anti-glycan response (comprising both IgM and class-switched antibody isotypes) appears non-protective (Hewitson et al., 2011). Glycan immunogenicity in the absence of overt anti-parasite effects is consistent with glycan “gimmickry”, whereby (potentially antigenic) helminth sugars actively manipulate the host immune response to prevent immunity (van Die and Cummings, 2010, Prasanphanich et al., 2013). Because of this, in characterising the overall glycan repertoire within HES, we also focussed on two main antigenic sugars in *H. polygyrus* infection, Glycans A and B.

The global glycan profile of HES reveals extensive and varied *O*-glycosylation, together with relatively restricted forms of *N*-glycosylation. Nevertheless, our studies provide evidence for methylation of both *O*- and *N*-linked glycans. One of the major sugars released from HES following β-elimination is a tri-substituted reducing HexNAc with three linked hexoses (H_3_N_1_ following sample permethylation). However, this is not detected in the (non-permethylated) hydrazine-released glycan pool, and instead a HexNAc with two bound hexoses and a 176 Da molecular species is seen. Whilst this could represent either a methylated hexose or a hexuronic acid, we view the latter as unlikely since we failed to detect a permethylated hexuronic acid in the β-elimination material. Additionally, the presence of methylated fucoses in the enzymatically released *N*-glycans (as well as in the hydrazinolysis released *O*-glycans) provides further evidence for this modification Glycan methylation has previously been found in bacteria, fungi and plants (Staudacher, 2012), as well as both parasitic (Khoo et al., 1991) and free-living (Guérardel et al., 2001, Haslam and Dell, 2003, Cipollo et al., 2005) nematodes, although multiple methylation of a single monosaccharide as found in *H. polygyrus* has only previously been reported in the deep sea annelid worm *Alvinella pompejana* (Talmont and Fournet, 1991).

Our data suggest that Glycan A represents a methylated di- or trisaccharide where the methyl group is present on a hexose residue. Glycan A is an immunodominant *O*-linked glycan coupled to three highly expressed proteins, VAL-1, 2 and 5, which all contain two CAP domains (Pfam00188) bridged by a glycosylated serine/threonine linker region (Hewitson et al., 2011). The isolation of a reactive Glycan A fraction by RP-chromatography required hydrazinolysis as methods employing permethylation would destroy the epitopic structure of the antigen, and to allow printing a novel *O*-glycan array for screening fractions. Whilst this approach extends that taken previously with *N*-linked and *O*-linked glycans and glycolipids in schistosome-infected individuals (van Diepen et al., 2012a, van Diepen et al., 2015), we were concerned that hydrazinolysis and subsequent labelling of the reducing sugar might also destroy *O*-linked epitopes, which encompass core residues, rather than antennary structural motifs common for *N*-linked glycans or larger *O*-glycans observed in schistosomes. Nevertheless, anti-Glycan A mAb bound to a single RP fraction (F20), as did polyclonal antibodies from immune mice. This reactive fraction contains methylated di- and tri-saccharides. Interestingly, it was previously found that methylated hexoses are also immunodominant antibody targets following infection with the nematode *Toxocara canis* (Schabussova et al., 2007)

Our work also provides a structural basis for antigenicity of Glycan B. This second immunogenic epitope is expressed on two components in HES, a heterodispersed high molecular weight antigen and a ∼65 kDa molecule, neither of which stained well with silver, suggestive of low protein content (Hewitson et al., 2011). Using a mammalian glycan array we now show that anti-Glycan B antibodies bind strongly to sulphated LDN motifs. Similar binding was also observed to LDN with α2,3-linked sialic acid, a monosaccharide generally absent from helminths (Johnston et al., 2009) and not found in this study. However, LDN-like structures were not observed following β-elimination or hydrazinolysis. This, coupled with the high molecular weight diffuse character of the major Glycan B-bearing antigen, suggests that the target is a GAG-containing polysaccharide of molecular mass greater than the detection range of the MS analyses (Yamada et al., 1999, Toyoda et al., 2000).

*Heligmosomoides polygyrus* glycans have previously been implicated in the immunosuppressive effects of this parasite, since heat denatured HES is able to prevent allergic lung inflammation (McSorley et al., 2012) and TLR-dependent DC activation (Massacand et al., 2009). Due to the novel nature of Glycan A, we tested whether it is an immunomodulatory structure by administering the naturally glycosylated protein VAL-2 to mice undergoing allergic airway sensitisation. However, our results showed that despite its immunodominance, Glycan A is not immunomodulatory in this setting, as neither native nor heat-denatured Glycan A-bearing VAL-2 reduces lung eosinophilia. The immunogenicity of Glycan A in the absence of overt immunosuppressive ability would be consistent with it fulfilling a diversionary role in generating ineffective antibodies. Alternative biochemical separation and affinity purification approaches are now being taken to identify the heat-stable immunomodulatory components in HES, which may or may not be carbohydrate in character.

Taken together, our results establish that *H. polygyrus* secretes a number of *O*-linked glycoconjugates, with marked glycan heterogeneity evident even on a single glycoprotein. Thus, if Glycan A is elaborated by the parasite as a decoy, this may have evolved to permit other glycans to function as modulators with minimal interference from host antibodies. Functional roles for these many other glycoconjugates are yet to be explored, although a number of potential interactions with the host can be envisaged. For example, glycans from other helminth species can, through their ability to ligate host lectin receptors such as C-type lectins (CTL) and galectins expressed by innate cells (e.g. DCs and macrophages), direct the host immune response (Prasanphanich et al., 2013). Thus, LeX-containing glycoconjugates similar to those produced by schisosomes can induce regulatory B cells and macrophages (Velupillai and Harn, 1994, Atochina et al., 2001, Atochina et al., 2008, Terrazas et al., 2001), and delay transplant rejection (Dutta et al., 2010) as well as the development of insulin resistance (Bhargava et al., 2012). LeX containing schistosome glycans have also been shown to have a key role in Th2 induction (Okano et al., 1999), and are required for mannose receptor-mediated internalisation of the ribonuclease omega-1, a pre-requisite for its Th2 skewing ability (Everts et al., 2012). Although these precise structures are not expressed by *H. polygyrus*, similar interactions through one or more of the many host pathogen pattern recognition receptors may prove to be important in the ability of this parasite to establish in the mammalian host.

## Figures and Tables

**Table 1:** Comparison of *O*-glycans released from *Heligmosomoides polygyrus* excretory–secretory products by hydrazinolysis and reductive β-elimination. *O*-glycans were liberated from *Heligmosomoides polygyrus* excretory–secretory products by hydrazinolysis as detailed in Section 2.6, labelled with anthranilic acid, and fractionated by reverse-phase liquid chromatography (RP-LC). Fractions were analysed by LC–MS/MS with glycans detected as [M+H]^+^. Compositions are based on *m*/*z* values and MS/MS fragmentation patterns. Equivalent permethylated glycans obtained by β-elimination (β-elim.) are also indicated.

LC-fraction	LC–MS [M+H]^+^	Composition	β-Elim. generated permethylated equivalent detected at	Notes
14	505.4	H_1_N_1_-AA	*m*/*z* 512.6 [M+H]^+^	
14	667.2	H_2_N_1_-AA	*m*/*z* 738.5 [M+Na]^+^	
14	708.5	H_1_N_2_-AA	*m*/*z* 779.6 [M+Na]^+^	Only found in VAL-2
15	505.4	H_1_N_1_-AA	*m*/*z* 512.6 [M+H]^+^	
15	843.8	meH_1_H_2_N_1_-AA	*m*/*z* 942.5 [M+Na]^+^	With me-hex (Δ176)
16	505.4	H_1_N_1_-AA	*m*/*z* 512.6 [M+H]^+^	
16	519.6	meH_1_N_1_-AA	*m*/*z* 512.6 [M+H]^+^	With me-hex (Δ176)
16	681.8	meH_1_H_1_N_1_-AA	*m*/*z* 738.5 [M+Na]^+^	With me-hex (Δ176)
16	772.8	F_1_H_3_-AA	*m*/*z* 841.5 [M+Na]^+^	Hexose at reducing end
16	843.8	meH_1_H_2_N_1_-AA	*m*/*z* 942.5 [M+Na]^+^	With me-hex (Δ176)
17	505.4	H_1_N_1_-AA	*m*/*z* 512.6 [M+H]^+^	
17	546.5	N_2_-AA		
17	681.8	meH_1_H_1_N_1_-AA	*m*/*z* 738.5 [M+Na]^+^	With me-hex (Δ176)
17	772.8	F_1_H_3_-AA	*m*/*z* 841.5 [M+Na]^+^	Hexose at reducing end
18	546.5	N_2_-AA		
18	610.2	F_1_H_2_-AA		Hexose at reducing end
19	546.5	N_2_-AA		
19	610.2	F_1_H_2_-AA		Hexose at reducing end
19	681.8	meH_1_H_1_N_1_-AA	*m*/*z* 738.5 [M+Na]^+^	With me-hex (Δ176)
19	709.6	H_1_N_1_-AA + Δ204		With Δ204 mass suggesting trimethyl-hex
19	756.6	F_2_H_2_-AA		Hexose at reducing end
20	519.6	meH_1_N_1_-AA	*m*/*z* 512.6 [M+H]^+^	With me-hex (Δ176)
20	681.8	meH_1_H_1_N_1_-AA	*m*/*z* 738.5 [M+Na]^+^	With me-hex (Δ176)
21	519.6	meH_1_N_1_-AA	*m*/*z* 512.6 [M+H]^+^	WITH me-hex (Δ176)
21	681.8	meH_1_H_1_N_1_-AA	*m*/*z* 738.5 [M+Na]^+^	With me-hex (Δ176)
21	813.2	F_1_H_2_N_1_-AA	*m*/*z* 912.5 [M+Na]^+^	
21	854.7	F_1_H_1_N_2_-AA	*m*/*z* 953.5 [M+Na]^+^	
22	547.2	N_1_-AA + Δ204		Δ204 mass suggesting trimethyl-hex
22	651.1	F_1_H_1_N_1_-AA	*m*/*z* 708.4 [M+Na]^+^	
23	448.4	F_1_H_1_-AA		Hexose at reducing end
23	477.8	H_1_meH_1_-AA		With me-hex at reducing end
24	448.4	F_1_H_1_-AA		Hexose at reducing end
25	462.5	meF_1_H_1_-AA		With me-fuc (Δ176), hexose at reducing end
26	462.5	meF_1_H_1_-AA		With me-fuc (Δ176), hexose at reducing end
	462.5	F_1_meH_1_-AA		With me-hex at reducing end
26	490.4	H_1_-AA + Δ188		Δ188 mass suggesting trimethyl-fuc, hexose at reducing end
	490.4	F_1_(Δ204)-AA		*m*/*z* 344 suggesting trimethyl-hex at reducing end

VAL-2, venom allergen/*Ancylostoma* secreted protein-like; me-hex, single methylation of hexose; me-fuc, single methylation of fucose; trimethyl-fuc, trimethyl-fucose; trimethyl-hex, trimethyl-hexose.

## Appendix A Supplementary data

Supplementary Figs. S1 and S2 Fig. S1. Competition ELISA reveals anti-Glycan A monoclonal antibodies (mAbs) bind a common antigen with a hierarchy of reactivity. Binding of biotinylated anti-Glycan A mAb clones (A) 2–2, (B) 3–28, (C) 2–12, (D) 3–29, (E) 3–11, (F) 2–13, (G) 3–8, (H) 2–62, (I) 3–40, (J) 13.1, (K) 3–42 and (L) 3–55 to excretory–secretory products of *Heligmosomoides polygyrus* (HES). Binding of biotinylated mAb was detected with streptavidin horseradish peroxidase (HRP). ELISAs were performed in the absence of additional antibodies (−), or after pre-incubation with an excess (xs) of the following competing unlabelled antibodies; control (ctrl) IgM mAb, anti-glycan (anti-gly) B mAb clone 9.1.3, or the 12 anti-glycan A mAb. Competitive binding between a particular biotinylated antibody and its unlabelled counterpart are shown in red. Antibodies are ordered by increasing binding affinity. Biotinylation of anti-glycan B mAbs ablated antibody reactivity, preventing a similar approach for these antibodies (data not shown). Fig. S2. Fragmentation of trisubstituted HexNAc glycan species H_3_N_1_. LC–MS/MS fragmentation spectrum of prominent glycan H_3_N_1_ excretory-secretory products of *Heligmosomoides polygyrus* (HES) observed at *m*/*z* [M+H]^+^ 920.9 ([M+Na]^+^ 942.5 in Fig. 3A) following β-elimination and glycan permethylation. Mass difference of 32 represents loss of methanol. HexNAc, square; hexose, circle; R, reduced end.
