# Supplementary material for: Novel O-linked methylated glycan antigens decorate secreted immunodominant glycoproteins from the intestinal nematode Heligmosomoides polygyrus
Source: Int J Parasitol. 2016 Mar;46(3):157–70. doi: 10.1016/j.ijpara.2015.10.004 (PMC4776704; doi:10.1016/j.ijpara.2015.10.004)
Supplement: Supplementary Figs. S1 and S2 — Fig. S1. Competition ELISA reveals anti-Glycan A monoclonal antibodies (mAbs) bind a common antigen with a hierarchy of reactivity. Binding of biotinylated anti-Glycan A mAb clones (A) 2–2, (B) 3–28, (C) 2–12, (D) 3–29, (E) 3–11, (F) 2–13, (G) 3–8, (H) 2–62, (I) 3–40, (J) 13.1, (K) 3–42 and (L) 3–55 to excretory–secretory products of Heligmosomoides polygyrus (HES). Binding of biotinylated mAb was detected with streptavidin horseradish peroxidase (HRP). ELISAs were performed in the absence of additional antibodies (−), or after pre-incubation with an excess (xs) of the following competing unlabelled antibodies; control (ctrl) IgM mAb, anti-glycan (anti-gly) B mAb clone 9.1.3, or the 12 anti-glycan A mAb. Competitive binding between a particular biotinylated antibody and its unlabelled counterpart are shown in red. Antibodies are ordered by increasing binding affinity. Biotinylation of anti-glycan B mAbs ablated antibody reactivity, preventing a similar approach for these antibodies (data not shown). Fig. S2. Fragmentation of trisubstituted HexNAc glycan species H3N1. LC–MS/MS fragmentation spectrum of prominent glycan H3N1 excretory-secretory products of Heligmosomoides polygyrus (HES) observed at m/z [M+H]+ 920.9 ([M+Na]+ 942.5 in Fig. 3A) following β-elimination and glycan permethylation. Mass difference of 32 represents loss of methanol. HexNAc, square; hexose, circle; R, reduced end. [file mmc1.pptx]

## Slide 1
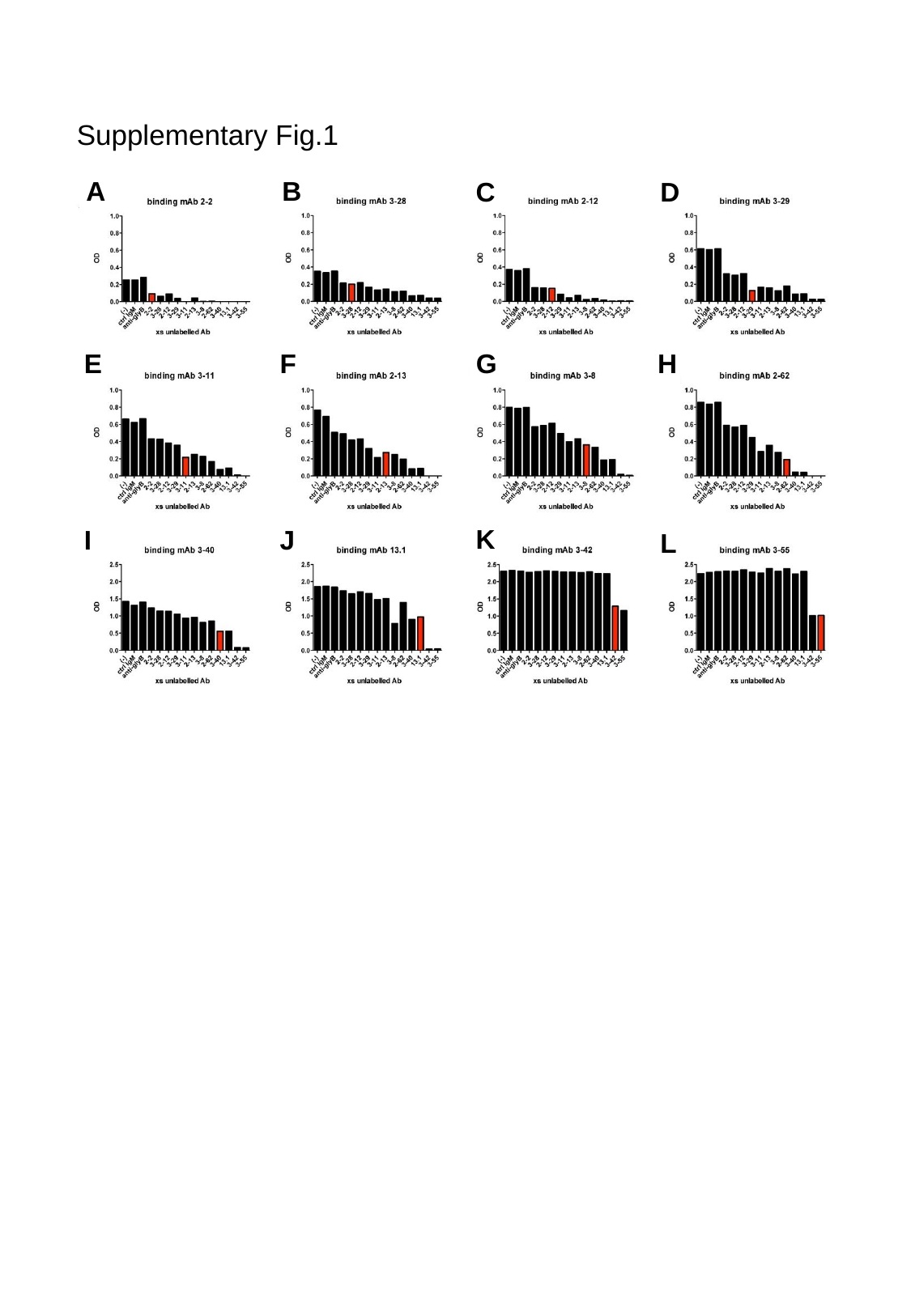

Supplementary Fig.1
A
B
C
D
E
F
G
H
K
I
J
L

## Slide 2
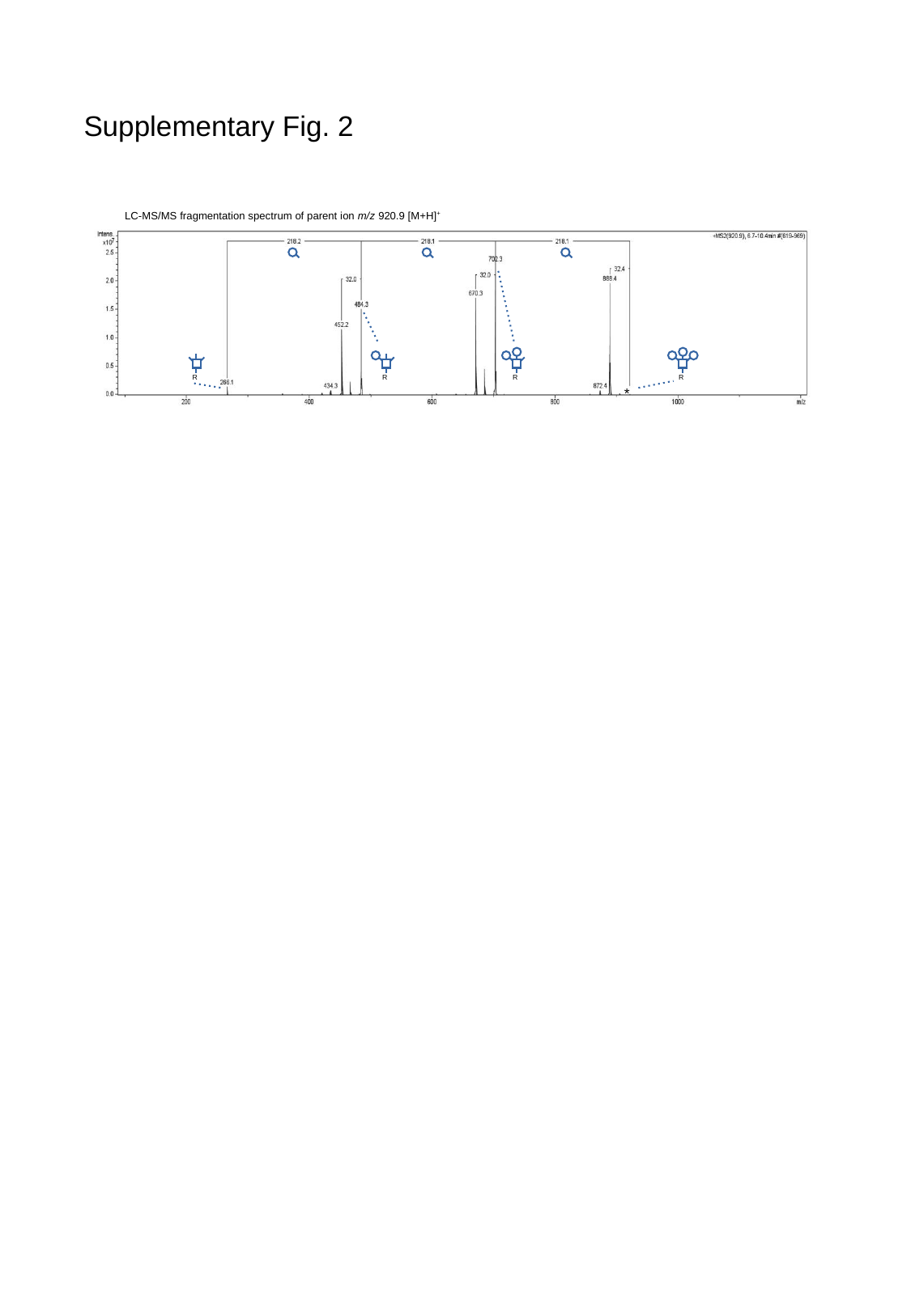

Supplementary Fig. 2
LC-MS/MS fragmentation spectrum of parent ion m/z 920.9 [M+H]+
R
R
R
R
*
